# Enhanced Electron Correlation and Significantly Suppressed Thermal Conductivity in Dirac Nodal‐Line Metal Nanowires by Chemical Doping

**DOI:** 10.1002/advs.202204424

**Published:** 2022-11-27

**Authors:** Amanda L. Coughlin, Zhiliang Pan, Jeonghoon Hong, Tongxie Zhang, Xun Zhan, Wenqian Wu, Dongyue Xie, Tian Tong, Thomas Ruch, Jean J. Heremans, Jiming Bao, Herbert A. Fertig, Jian Wang, Jeongwoo Kim, Hanyu Zhu, Deyu Li, Shixiong Zhang

**Affiliations:** ^1^ Department of Physics Indiana University Bloomington IN 47405 USA; ^2^ Department of Mechanical Engineering Vanderbilt University Nashville TN 37235 USA; ^3^ Department of Physics Incheon National University Incheon 22012 Korea; ^4^ Electron Microscopy Center Indiana University Bloomington IN 47405 USA; ^5^ Department of Mechanical and Materials Engineering University of Nebraska Lincoln NE 68588 USA; ^6^ Center for Integrated Nanotechnologies, MPA Division Los Alamos National Laboratory Los Alamos 87545 United States; ^7^ Department of Electrical and Computer Engineering and Texas Center for Superconductivity (TcSUH) University of Houston Houston TX 77204 USA; ^8^ Department of Physics Virginia Tech Blacksburg VA 24061 USA; ^9^ Department of Materials Science and NanoEngineering Rice University Houston TX 77005 USA

**Keywords:** 5d transition metal oxides, electron correlation, iridium dioxide, thermal conductivity

## Abstract

Enhancing electron correlation in a weakly interacting topological system has great potential to promote correlated topological states of matter with extraordinary quantum properties. Here, the enhancement of electron correlation in a prototypical topological metal, namely iridium dioxide (IrO_2_), via doping with 3d transition metal vanadium is demonstrated. Single‐crystalline vanadium‐doped IrO_2_ nanowires are synthesized through chemical vapor deposition where the nanowire yield and morphology are improved by creating rough surfaces on substrates. Vanadium doping leads to a dramatic decrease in Raman intensity without notable peak broadening, signifying the enhancement of electron correlation. The enhanced electron correlation is further evidenced by transport studies where the electrical resistivity is greatly increased and follows an unusual $\sqrt{T}$ dependence on the temperature (*T*). The lattice thermal conductivity is suppressed by an order of magnitude via doping even at room temperature where phonon‐impurity scattering becomes less important. Density functional theory calculations suggest that the remarkable reduction of thermal conductivity arises from the complex phonon dispersion and reduced energy gap between phonon branches, which greatly enhances phase space for phonon–phonon Umklapp scattering. This work demonstrates a unique system combining 3d and 5d transition metals in isostructural materials to enrich the system with various types of interactions.

## Graphical abstract

Enhancement of electron correlations in topological metal IrO_2_ nanowires is achieved by doping with 3d transition metal vanadium, as evidenced by a dramatic decrease in Raman intensity and unusual temperature dependence of resistivity. The lattice thermal conductivity is suppressed by an order of magnitude via doping due to the complex phonon dispersion and reduced energy gap between phonon branches.

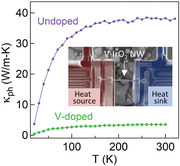

## 1 Introduction

Transition metal oxides (TMOs) have been the subject of extensive studies for many decades owing to their fascinating physical and chemical properties.^[^ ^1^, ^2^, ^3^, ^4^, ^5^ ^]^ In particular, 3d TMOs (e.g., cuprates and manganites) have shown a broad spectrum of intriguing phenomena, including metal–insulator transitions,^[^ ^1^, ^6^ ^]^ high‐temperature superconductivity,^[^ ^7^, ^8^ ^]^ colossal magnetoresistance,^[^ ^9^, ^10^ ^]^ and multiferroicity.^[^ ^11^, ^12^ ^]^ A fundamental driving force for these exotic phenomena is the strong correlation between the 3d electrons due to the compact orbitals that they occupy.^[^ ^1^, ^13^, ^14^ ^]^ The spin–orbit coupling (SOC), however, is rather weak in many 3d TMOs because of the low atomic number of 3d transition metal elements. On the other hand, 5d TMOs, such as iridates, possess stronger SOC due to the considerably higher atomic number, as demonstrated experimentally by X‐ray absorption spectroscopy.^[^ ^15^ ^]^ The strong SOC gives rise to a variety of intriguing topological and magnetic states, namely topological semimetals,^[^ ^16^, ^17^ ^]^ spin–orbit coupled Mott insulators,^[^ ^18^, ^19^ ^]^ and quantum spin liquids.^[^ ^20^, ^21^, ^22^, ^23^ ^]^ The electron correlation in 5d TMOs, however, is notably weaker than in 3d TMOs due to the more spatially extended 5d orbitals.

Enhancing correlations in a spin–orbit coupled system has great potential to promote correlated topological states of matter and new physical phenomena emerging from the interplay of electron interactions and SOC.^[^ ^4^, ^24^ ^]^ While it has been shown that the existence of strong SOC in some iridates (e.g., Sr_2_IrO_4_)^[^ ^18^, ^19^ ^]^ could enhance the effects of correlation, this enhancement is lacking in many others. A prominent example is the binary iridium dioxide (IrO_2_) in which the electron correlation is negligible despite the strong SOC.^[^ ^25^, ^26^ ^]^ Recent density functional theory (DFT) calculations, along with angle‐resolved photoemission spectroscopy (ARPES) studies, have demonstrated that IrO_2_ is a topological metal with Dirac nodal lines protected by the non‐symmorphic symmetry of its rutile crystal structure.^[^ ^27^, ^28^, ^29^ ^]^ Furthermore, a large orientation‐dependent spin Hall effect was detected in IrO_2_,^[^ ^30^, ^31^ ^]^ which was attributed to the SOC‐induced band anti‐crossing.^[^ ^27^ ^]^ In addition to electronic and spintronic properties, IrO_2_ also exhibits interesting thermal properties, where phonon transport plays a dominant role in the total thermal conductivity,^[^ ^32^ ^]^ unlike many other metals. This large lattice contribution arises from the strong interatomic bonding and large atomic mass difference between iridium and oxygen, which competes with the suppression induced by strong electron–phonon interactions.^[^ ^32^ ^]^ Beyond fundamental studies, IrO_2_ has technological importance in various areas including water splitting,^[^ ^33^, ^34^ ^]^ ferroelectric memories,^[^ ^35^ ^]^ and electrochemical devices.^[^ ^30^, ^36^ ^]^

**Figure 1:**
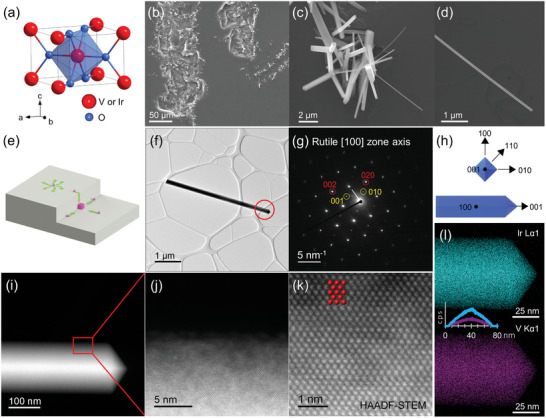
a) A schematic of a rutile tetragonal crystal structure. b–d) SEM images of Ir_1 −_ *_x_* V*_x_* O_2_ nanowires grown in the scratched regions. e) A schematic illustrating the diffusion of atoms in an isotropic manner when isolated away from edges, or toward the edges when in the vicinity. f) A low‐magnification TEM image and g) the corresponding diffraction pattern along the [100] zone axis. h) A schematic showing the geometry of an Ir_1 −_ *_x_* V*_x_* O_2_ nanowire. i–k) HAADF STEM images, and l) XEDS maps of Ir and V elements in an Ir_1 −_ *_x_* V*_x_* O_2_ nanowire (*x* ≈ 0.29) with the XEDS line scans across the diameter of the nanowire (inset). The intensity of Ir and V decreases further from the center of the nanowire due to the thickness varying across the diameter when the electron beam is not perpendicular to the nanowire surfaces.

In this work, we doped weakly‐interacting IrO_2_ nanowires with 3d transition metal vanadium to enhance electron correlations in this spin–orbit coupled system. Vanadium was chosen as the dopant over other 3d transition metals for the following reasons: 1) its binary oxide VO_2_ can also be stabilized in a rutile phase (**Figure** **1**a),^[^ ^37^ ^]^ allowing for high dopant levels in IrO_2_ while still maintaining its rutile crystal structure; 2) strong electron correlation has been clearly demonstrated in VO_2_ and it plays a significant role in determining the electronic and optical properties.^[^ ^38^ ^]^ Compared to bulk materials in which the study of true microscopic properties is often challenging due to inhomogeneity or complex domain structures, single‐crystalline nanowires offer an excellent platform to probe the intrinsic properties at the microscopic scale.^[^ ^39^, ^40^, ^41^, ^42^, ^43^ ^]^ As such, we synthesized single‐crystalline V‐doped IrO_2_ (Ir_1 −_ *_x_* V*_x_* O_2_) nanowires by chemical vapor deposition (CVD) (Figure S1, Supporting Information). Through Raman scattering, electrical and thermal transport measurements, and in correlation with DFT calculations, we demonstrated the enhancement of electron correlation and a remarkable suppression of thermal conductivity in the nanowires upon V‐doping.

## 2 Results and Discussions

Ir_1 −_ *_x_* V*_x_* O_2_ nanowires were grown on Si substrates via a CVD process in which IrO_2_ and VO_2_ powders were used as precursors. The previous synthesis of undoped nanowires suggests that IrO_2_^[^ ^44^, ^45^ ^]^ and VO_2_^[^ ^46^, ^47^, ^48^, ^49^ ^]^ prefer to grow in O_2_ and Ar (or other inert gas such as He) atmospheres, respectively; thus, a combination of O_2_ and Ar carrier gasses were used to realize the growth of doped Ir_1 −_ *_x_* V*_x_* O_2_ nanowires in this work. The Si substrates were scratched with a diamond tip pen to create fresh and rough surfaces, which tend to promote nanowire growth. Indeed, as shown in Figure 1b,c, a high density of nanowires/nanorods are grown within these scratched areas, in contrast to a previous growth where plate‐ or particle‐like structures were formed.^[^ ^50^ ^]^ The nanowires grown within the scratch are typically smooth, straight, have rectangular cross‐sections, and have lengths up to tens of micrometers and diameters down to ~≈40 nm (Figure 1d, as an example).

The density of the nanowires in these areas is typically higher than in the nearby flat regions (Figure 1b and Figure S2, Supporting Information). This is due to the atoms preferring to diffuse to the edges within the scratched regions because of the increased area of rougher surfaces, as well as the ability to interact with more than one surface at a time which lowers the formation energy of the nucleation sites, relative to flat surfaces.^[^ ^51^, ^52^, ^53^ ^]^ As depicted in Figure 1e, atoms that have landed on the flat substrate surface will diffuse in an isotropic manner along the surface, while those near edges will be biased toward the corners or edges where they can interact with more than one surface.^[^ ^54^, ^55^, ^56^ ^]^ Thus, more atoms diffusing to the edges in the scratches will lead to an increased number of nucleation sites from which a higher density of nanowires will grow, compared to the flat substrate areas. Nanowires grown within the flat regions are generally more tapered and partially embedded in the substrate near the “root” as the diffused atoms wet the smooth surface which encourages growth along the substrate (Figure S3, Supporting Information).

The Ir_1 −_ *_x_* V*_x_* O_2_ nanowires are single‐crystalline with a tetragonal rutile structure and a growth direction along [001]. The single‐crystallinity is demonstrated by the clear, isolated spots in the diffraction pattern (Figure 1g), which can be indexed to the rutile‐structured [100] zone axis. The growth direction of the nanowire is clearly along the [001] direction, and in combination with the known zone axis, the main side facets of the nanowire belong to the {110} planes (Figure 1h), the most stable surface orientations in both rutile VO_2_^[^ ^57^ ^]^ and IrO_2_^[^ ^58^ ^]^ structures. Extinction spots, or forbidden spots, are present in the diffraction pattern, as indicated by the yellow circles in Figure 1g; their appearance is due to dynamical scattering events when the electron beam is re‐diffracted multiple times.^[^ ^59^ ^]^ High‐angle annular dark‐field (HAADF) scanning transmission electron microscopy (TEM) images (Figure 1i–k) show clear lattice fringes, further confirming the high crystalline quality of the nanowires. Additionally, Figure 1k shows distinct metal columns (highlighted in red), indicating that the V atoms indeed substitute the Ir atoms as opposed to occupying interstitial sites between metal columns. As shown in the X‐ray energy‐dispersive spectroscopy (XEDS) (Figure 1l), the nanowire is uniformly composed of Ir and V elements (in spite of thickness variation), and a vanadium doping concentration of up to ≈30% was achieved through the CVD process.

**Figure 2:**
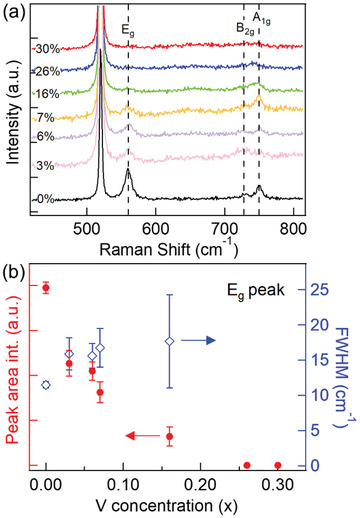
a) Raman spectra of Ir_1 −_ *_x_* V*_x_* O_2_ nanowires of varied V concentrations and b) the integrated area intensity and full‐width‐half‐maximum of the E_g_ mode as a function of V concentration determined by SEM‐XEDS (Sections S5 and S6, Supporting Information). All Raman measurements were performed at the same laser power and all of the nanowires were of similar widths with a variation of less than 10%.

We found evidence of enhanced electron correlation by conducting Raman scattering studies on the Ir_1 −_ *_x_* V*_x_* O_2_ nanowires at different doping concentrations. Both tetragonal rutile IrO_2_ and VO_2_ show finite optical conductivity at the Raman excitation energy of 532 nm (2.33 eV).^[^ ^60^, ^61^ ^]^ The resonant Raman scattering cross‐section is approximately inversely proportional to *γ* ^4^, where *γ* is the lifetime of the intermediate electronic excitations.^[^ ^62^ ^]^ The IrO_2_ exhibits strong Raman peaks at ≈561, 728, and 752 cm^−1^ belonging to the E_g_, B_2g_, and A_1g_ modes, respectively.^[^ ^63^ ^]^ In contrast, rutile VO_2_ has no observable Raman peaks due to its very short excitation lifetime but only weak and broad bands centered near ≈230 and ≈450 cm^−1^.^[^ ^64^ ^]^ As shown in **Figure** **2**a, the Raman intensity of Ir_1 −_ *_x_* V*_x_* O_2_ nanowires dramatically decreases as the vanadium concentration *x* increases, accompanied by a slight broadening of the peak width. Quantitatively, the integrated area intensity of the E_g_ mode is reduced by 34% with *x* as small as 0.03 and almost vanishes at *x* ≈ 0.16 (Figure 2b). The A_1g_ mode of the rutile phase is still detectable up to *x* ≈ 0.26 and no new Raman peaks appear, ruling out the possibility of a structural phase transition in this doping range. Therefore, the significant suppression of Raman intensity strongly suggests a reduction of electronic excitation lifetime upon doping, which could result from the enhanced impurity scattering and/or electron correlation. Although impurity scattering can potentially reduce electronic excitation lifetime, it is expected to broaden the Raman peak but not significantly decrease the integrated intensity in the absence of electron correlation.^[^ ^65^ ^]^ Indeed, previous Raman measurement of Cr‐doped IrO_2_ shows that the characteristic peaks are still clearly visible with some broadening at a dopant concentration as high as *x* = 0.4.^[^ ^66^ ^]^ In this Cr‐doped system, doping induces impurity scattering without notably enhancing electron correlation as CrO_2_ itself is demonstrated to be weakly correlated.^[^ ^67^, ^68^ ^]^ Therefore, the drastic decrease in Raman intensity observed in our V‐doped system strongly suggests the enhancement of electron correlation, which suppresses the electronic lifetime.

**Figure 3:**
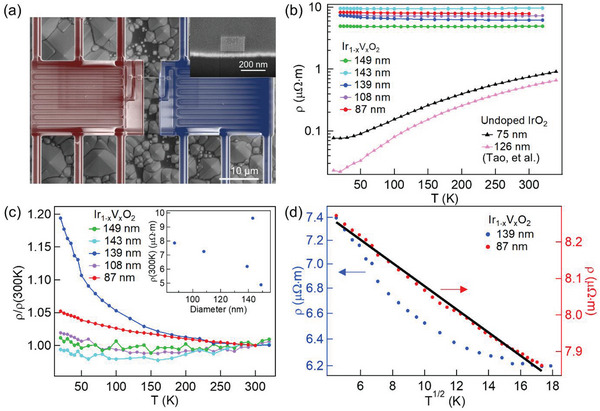
a) An SEM image of a typical measurement device and an inset showing the rectangular cross‐section of the nanowire. b) The resistivities of the Ir_1 −_ *_x_* V*_x_* O_2_ (*x* = 0.29 ± 0.01) and undoped IrO_2_ nanowires^[^ ^32^ ^]^ and (c) the normalized resistivities of the Ir_1 −_ *_x_* V*_x_* O_2_ nanowires as a function of temperature. The inset shows the resistivities at 300 K as a function of nanowire diameter. d) The resistivities of the 139 and 87 nm nanowires as a function of $\sqrt{T}$. The black solid line is a linear fit of the resistivity.

We further carried out temperature‐dependent electrical resistivity, Seebeck coefficient, and thermal conductivity measurements to understand the influence of doping. The dopant concentrations of the nanowires, as characterized by XEDS studies, are *x* = 0.29 ± 0.01 (Table S1, Supporting Information), which is close to the value (*x* = 0.25) used in our DFT calculations as discussed below. **Figure** **3**a shows a typical measurement device in which an individual nanowire was placed between two suspended membranes with integrated platinum coils serving as resistance heaters and thermometers. Extra electrodes are embedded to allow for 4‐point electrical resistivity measurements.^[^ ^69^, ^70^, ^71^ ^]^ As shown in the inset of Figure 3a, the nanowires have a nearly rectangular cross‐section and were labeled with their hydraulic diameter (*D*_h_), which is defined as four times the reciprocal of the surface‐area‐to‐volume ratio.

The Ir_1 −_ *_x_* V*_x_* O_2_ (*x* = 0.29 ± 0.01) nanowires are orders of magnitude more resistive than the undoped IrO_2_, as shown in Figure 3b. Since the total density of states (DOS) calculated by DFT does not show a significant change upon doping (Figure S9, Supporting Information), it is reasonable to believe that the charge carrier density is not strongly affected by doping and the dramatic increase of resistivity is due to a reduction of mobility, which could result from the enhanced electron correlation, as well as impurity scattering. For the undoped case, the 75 nm diameter nanowire has a higher resistivity than the 126 nm nanowire, which was attributed to the enhanced surface scattering of electrons and possibly a varied density of point defects.^[^ ^32^ ^]^ The five measured Ir_1 −_ *_x_* V*_x_* O_2_ nanowires have diameters varying from 87 to 149 nm with resistivities that follow the same diameter dependence observed in the undoped nanowires, with the exception of the 143 nm wire (inset of Figure 3c). The diameter dependence again suggests that surface boundary scattering plays a role in charge transport. The exception of the 143 nm nanowire may result from a slightly different vanadium concentration although such a small variation in composition was not captured by XEDS due to its relatively large measurement uncertainty.

In contrast to undoped IrO_2_, which is metallic from 300 down to 20 K,^[^ ^32^ ^]^ Ir_1 −_ *_x_* V*_x_* O_2_ (*x* = 0.29 ± 0.01) nanowires typically show a non‐metallic behavior in a certain temperature range as evidenced by the increase of resistivity upon cooling (Figure 3c). It is worth noting, however, that the change of resistivity with temperature is rather small and the standard thermal activation model for semiconductors can hardly describe such a weak temperature dependence (Figure S10, Supporting Information, where our analysis focuses on the 87 and 139 nm nanowires, which have the highest signal to noise ratio). This is consistent with the DFT calculations, which suggest that the system is a semimetal rather than a semiconductor (Figures S9 and S11, Supporting Information). We note that the upturn of resistivity at low temperatures in a metallic doped (disordered) system could arise from mechanisms including Anderson (strong) localization, the Kondo effect, weak localization, and a correction due to the effects of electron–electron interaction (EEI) on the DOS. Electron wave functions can undergo Anderson localization due to a disordered lattice potential in a system with impurities or defects.^[^ ^72^, ^73^, ^74^, ^75^ ^]^ Nevertheless, as discussed in Section S11, Supporting Information, the rather weak temperature dependence of resistivity again contrasts with a typical Anderson localization behavior where resistivity has a stretched exponential dependence on temperature according to the variable‐range‐hopping model.^[^ ^76^, ^77^, ^78^ ^]^

The conventional spin Kondo effect describes the scattering of conduction electrons by magnetic impurities and usually occurs in metals dilutely doped with magnetic impurities.^[^ ^79^, ^80^ ^]^ Since the host IrO_2_ is a paramagnetic metal with a non‐zero magnetic moment on each Ir^4+^ site, the spin Kondo effect is unlikely to occur in this dense magnetic system. We note, however, the orbital two‐channel Kondo effect (2CK) was recently proposed to explain the resistivity upturn below ≈20 K in oxygen‐deficient IrO_2_ nanowires.^[^ ^81^ ^]^ In brief, each oxygen vacancy can lead to a two‐fold degeneracy of the *d*_xz_ and *d*_yz_ orbitals around the nearest Ir ions. Such a degeneracy drives the orbital 2CK effect where the degenerate orbitals form a local pseudospin basis and the spin states of the conduction electrons act as a channel index.^[^ ^81^ ^]^ The 2CK is associated with strong electron correlation and manifests by a $\sqrt{T}$ dependence of resistivity increase upon cooling. As shown in Figure 3d, such a $\sqrt{T}$ dependence is observed in the entire temperature range in the 87 nm nanowire. For the 139 nm wire, the $\sqrt{T}$ dependence exists in a rather narrow range in the low temperature region. Since precise control of oxygen vacancies is challenging in a CVD process, the quantitative difference may arise from the different densities of oxygen vacancies between nanowires, as was observed in the undoped IrO_2_.^[^ ^81^ ^]^ As discussed in detail in Section S12, Supporting Information, magnetoresistance measurements at various temperatures between 5 and 200 K show that the magnetic field has a negligible influence on the resistivity (Figure S13, Supporting Information), consistent with the nonmagnetic nature of the orbital 2CK effect.^[^ ^81^ ^]^

Two other plausible mechanisms can lead to the mild increase of resistivity with decreasing temperature, both in moderate disorder: weak localization and the exchange/Hartree correction due to EEI. While the weak‐localization effect does not produce a $\sqrt{T}$ dependence of resistivity, direct EEI in a weakly disordered metal does, essentially due to its effect on the DOS.^[^ ^73^, ^74^, ^75^, ^81^, ^82^ ^]^ Although the EEI contribution was ruled out in the undoped IrO_2_ by analyzing the magnitude of resistivity changes;^[^ ^81^ ^]^ a similar analysis on the doped nanowires, however, cannot distinguish the contributions from the EEI and the 2CK effect (Section S13, Supporting Information). Interestingly, both the 2CK effect and the direct EEI correction provide evidence that the system possesses strong electron correlations, which is one of the main points in this work. It is worth noting that the persistence of non‐metallic behavior at high temperatures indicates a minimal effect of electron–phonon interactions on the charge transport. Indeed, as discussed in detail in Section S14, Supporting Information, the overall mean free path (*l*) estimated from the transport data and DFT calculations is on the order of 1 nm, which is significantly smaller than the estimated mean free path *l*_e − ph_ due to electron–phonon interactions.

**Figure 4:**
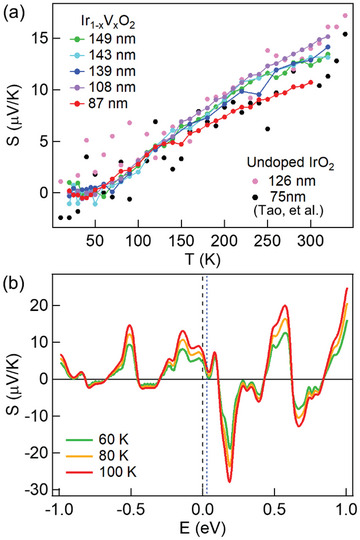
a) The measured Seebeck coefficients (*S*) of Ir_1 −_ *_x_* V*_x_* O_2_ (*x* = 0.29 ± 0.01) and undoped IrO_2_ nanowires^[^ ^32^ ^]^ of various diameters as a function of temperature and b) the calculated *S* of an Ir_0.75_V_0.25_O_2_ nanowire as a function of energy between 60 and 100 K. Due to the higher thermal conductivity and thus larger thermal conductance of the undoped IrO_2_ nanowires, the temperature difference across these undoped nanowires is much smaller compared to the doped nanowires. This leads to a smaller Seebeck voltage and thus a considerably larger experimental uncertainty for the Seebeck coefficient of the undoped IrO_2_ nanowires. The black dashed line denotes the Fermi energy of a stoichiometric sample in the DFT calculation, while the blue dotted line indicates the Fermi energy estimated from the comparison of experimental and calculated *S* values.

The Seebeck coefficients (*S*) of the doped nanowires are comparable to those of the undoped wires (**Figure** **4**a). Compared to heavy element‐based thermoelectric materials, the small *S* in oxides is often attributed to the relatively low electron mobility due to the more ionic bonding, the localization of charge carriers, and a strong scattering of carriers by optical phonons.^[^ ^83^ ^]^ For both the undoped and doped IrO_2_, the small *S* also results from the coexistence of electron and hole pockets (Figure S11, Supporting Information), the contribution of which cancels each other. The value of *S* decreases and trends toward zero as *T* decreases, in spite of the large experimental uncertainty in the undoped nanowire measurement due to their much higher thermal conductivity. Based on the DOS of Ir_0.75_V_0.25_O_2_ (Figure S9, Supporting Information), we further calculated the *S* at different temperatures as a function of Fermi energy (Figure 4b) and compared the results with the experimental values determined by averaging the measured *S* of five doped nanowires at each temperature. Below 50 K, the experimental *S* is negligible; it increases to 0.48 ​µV K^−1^ at 60 K, 1.45 µV K^−1^ at 80 K, and 2.65 µV K^−1^ at 100 K. A comparison with the calculation shows the Fermi energy is shifted only marginally to ≈+0.03 eV (Figure 4b), which may be due to the existence of n‐type point defects such as oxygen vacancies in the nanowires.

**Figure 5:**
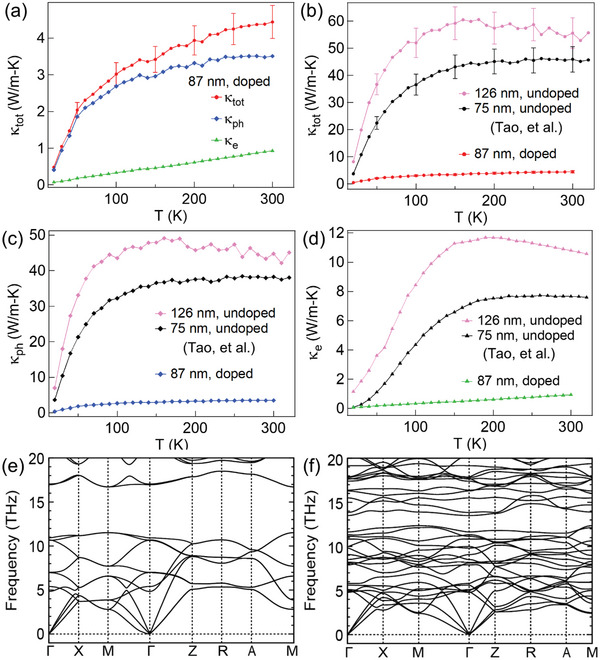
Thermal properties of Ir_1 −_ *_x_* V*_x_* O_2_ (*x* = 0.29 ± 0.01) nanowires. a) The total thermal conductivity of an 87 nm wire with the electronic and lattice contributions. The effect of V doping on b) the total thermal conductivity, c) lattice thermal conductivity, and d) electronic thermal conductivity compared to IrO_2_ nanowires.^[^ ^32^ ^]^ Phonon dispersion relation of e) undoped IrO_2_ and f) Ir_0.75_V_0.25_O_2_.

Unlike the Seebeck coefficient, thermal conductivity is greatly impacted by vanadium doping. **Figure** **5**a shows the thermal conductivity (*κ*_tot_) of an 87 nm diameter Ir_1−_ *_x_* V*_x_* O_2_ nanowire as an example, in comparison with the undoped samples (Figure 5b–d). The related lattice (*κ*_ph_) and electronic (*κ*_e_) contributions are plotted, with *κ*_e_ being estimated using the Wiedemann–Franz law and the Lorenz number (*L*) calculated by

(1) $$L=1.5+\exp\left[-\frac{\left|S\right|}{116}\right]$$

where *L* is in 10^−8^ W Ω K^−2^, and *S* is in µV K^−1^.^[^ ^84^ ^]^ *κ*_ph_ was obtained by subtracting *κ*_e_ from *κ*_tot_. Both electrons and phonons make non‐negligible contributions to *κ*_tot_ (Figure 5a). A drastic reduction of *κ*_ph_ by about one order of magnitude was observed in comparison to the undoped nanowires of similar sizes (Figure 5c). The *κ*_ph_ of the doped nanowire is even smaller than that of rutile VO_2_ nanowires.^[^ ^85^ ^]^ In addition, a monotonous increase in *κ*_ph_ with temperature was observed in the entire temperature range, unlike the undoped 126 nm diameter wire. Doping inevitably enhances impurity scattering, which reduces the lattice thermal conductivity. Previous studies of the doping effect on *κ*_ph_^[^ ^86^, ^87^, ^88^ ^]^ suggested up to one order of magnitude reduction in the thermal conductivity only at low temperatures where impurity scattering plays a dominant role. At room temperature, the reduction is usually much smaller as the major scattering mechanism shifts to phonon–phonon scattering.

To understand the drastic reduction in *κ*_ph_ at high temperatures, we calculated the phonon dispersions of undoped IrO_2_ and Ir_0.75_V_0.25_O_2_ using DFT calculations. As shown in Figure 5e,f, doping leads to a much more complex phonon dispersion with a reduction in the energy gap (from 5 to 1.4 THz) between different phonon branches. The more complex phonon dispersion is related to an increased number of atoms per primitive unit cell, as well as the lowering of symmetry in the cell, whereas the reduction in energy gap originates from the reduced mass ratio^[^ ^89^ ^]^ of the transition metal atom to the oxygen atom as vanadium is much lighter than iridium. With the more complex dispersion and reduced gap, more optical phonon modes are available, which increases the phase space for phonon–phonon Umklapp scattering.^[^ ^89^, ^90^, ^91^, ^92^ ^]^ As a result, the phonon mean free path and *κ*_ph_ are significantly reduced even at room temperature. In addition to doping, surface boundary scattering also plays a role in the thermal transport of nanowires. Figure S16, Supporting Information, plots the thermal conductivity of several nanowires, which in general decreases as the wire diameter decreases.

## 3 Conclusion

In summary, we demonstrated the CVD growth of single‐crystalline Ir_1 −_ *_x_* V*_x_* O_2_ nanowires with improved yield and morphology by creating surface roughness on substrates to increase nucleation sites and surface wetting. The Raman scattering study shows a dramatic decrease in Raman peak intensity upon vanadium doping, indicating a reduction of the electronic excitation lifetime by enhanced electron correlation. While DFT calculations suggest a metallic system, a non‐metallic behavior was experimentally observed in Ir_1 −_ *_x_* V*_x_* O_2_ (*x* = 0.29 ± 0.01) nanowires, which are orders of magnitude more resistive than the Dirac metal IrO_2_. The fitting of the temperature dependence of electrical resistivity suggests a possible 2CK effect and/or direct EEI, both of which describe systems with strong electron correlation. The Seebeck coefficient is limited by the co‐existence of electron and hole pockets, which contribute to opposite signs. The lattice thermal conductivity is suppressed by an order of magnitude upon vanadium doping even at room temperature where phonon‐impurity scattering becomes less important. Such a remarkable reduction in the doped nanowires is attributed to a more complex phonon dispersion with a significantly reduced energy gap between different phonon branches, rendering greatly enhanced phase space for phonon–phonon Umklapp scattering. Our work demonstrates a unique system that combines 3d and 5d transition metals in isostructural materials to enhance various types of interactions.

## 4 Experimental Section

### Nanowire Synthesis

V‐doped IrO_2_ (Ir_1 −_ *_x_* V*_x_* O_2_) nanowires were synthesized using a three‐zone quartz tube furnace via a CVD process (Figure S1, Supporting Information). Vanadium oxide (VO_2_) powder (Aldrich, ≥99% trace metals basis) was placed in an alumina boat in the center of the upstream zone 1 and iridium oxide (IrO_2_) powder (Alfa Aesar, 99.99% metals basis) was placed in the center of the furnace in zone 2. Four silicon substrates, ≈3 in. each in length, were placed one after another beginning immediately after the IrO_2_ powder boat, extending from ≈0.5 to ≈12.5 in. away from the center of zone 2 into downstream zone 3. An amorphous native oxide was expected to have formed non‐uniformly on the surface of the silicon substrate. A diamond‐tipped pen was used to scratch the substrates every ≈1 in., starting from ≈1.5 in. downstream from the IrO_2_ boat, which greatly promoted the growth and yield of nanowires. The pressure of the quartz tube was 900 Torr and a 40 sccm flow of Ar gas was introduced as the initial carrier gas during the ramping‐up segment of the growth until the growth temperatures of 600, 950, and 600 °C for zones 1, 2, and 3, respectively, were reached. During the soaking segment, O_2_ gas was introduced (in addition to the Ar gas) at a rate of 20 sccm. O_2_ was not flowed during the ramping segment as VO_2_ can easily be oxidized into V_2_O_5_, which was significantly more volatile than IrO_2_, and also to prevent the V vapor species from being introduced to the growth before the Ir vapor. Immediately after the growths, the furnace was turned off, the system was evacuated down to the base pressure of ≈1.5 Torr, the Ar flow was turned off, and the O_2_ flow rate was decreased to 2 sccm until the furnace was cooled down to room temperature.

### Characterizations

The morphologies of the Ir_1 −_ *_x_* V*_x_* O_2_ nanowires were characterized with a scanning electron microscope (SEM, Quanta FEI and Zeiss Auriga 60), and the structure and growth orientations were characterized via TEM (JEOL 3200 TEM), and the chemical composition of the nanowires determined with XEDS in the SEM, JEOL 3200 TEM, and NEOARM atomic resolution analytical electron microscope by JEOL. The Raman spectra were collected at room temperature with a Renishaw inVia confocal Raman microscope system with a 532 nm excitation laser with a nominal laser power of 50 µW and calibrated with a reference Si peak at 520 cm^−1^.

### Electrical and Thermal Transport Property Measurements

All transport property measurements, except the magnetoresistance measurement, were performed in a cryostat (Janis CCS‐400/204) operated under a high vacuum condition (<1  ×  10^−6^ mbar).^[^ ^69^, ^71^ ^]^ Thermal and electrical conductivities, as well as Seebeck coefficients of individual Ir_1 −_ *_x_* V*_x_* O_2_ nanowires were extracted using a well‐established micro‐thermal bridge method. During thermal conductance measurements, two radiation shields were applied to reduce the radiation loss and to achieve a better resolution, a Wheatstone bridge circuit configuration was adopted to increase the sensitivity for temperature measurements.^[^ ^70^ ^]^ A nanowire sample was placed between two adjacent suspended membranes with embedded serpentine platinum coils supported by 6 SiN*_x_* beams (Figure 3a). Each platinum coil, functioning as a resistance heater/thermometer, was electrically connected to four contact pads by Pt lines on the suspended beams, which enabled electrical joule heating and four‐point measurements of its resistance. During the measurement, a DC bias voltage applied to one of the resistors (*R*_h_) created Joule heating and increased the temperature (*T*_h_) of the heating membrane above the thermal bath temperature (*T*_0_). Part of the heat flowed through the nanowire to the other resistor (*R*_s_) and raised its temperature (*T*_s_). Solving the heat transfer equations of the whole system yielded the thermal conductance of the nanowire. The thermal conductivity can be derived after the length and cross‐sectional area of the nanowire was extracted from the microscopy characterizations. Two extra electrodes were patterned at the inner edges of each of the suspended membranes for electrical measurements. To minimize the contact thermal resistance between the sample and the two membranes and to facilitate the electrical and Seebeck measurements, local electron beam‐induced deposition (EBID, FEI Helios NanoLab G3) of Pt/C composites was performed at the contacts between the nanowire and Pt electrodes. Before the EBID process, reagent alcohol was also used to wet the wires (wetting treatment), and the evaporation of alcohol resulted in intimate contact between the wire and the suspended membranes.^[^ ^32^, ^93^, ^94^ ^]^ The Seebeck coefficient was extracted during the thermal measurements by monitoring the temperature difference Δ*T* and voltage difference Δ*V* (Stanford Research, SR560) across the sample (*S*_measured_ = −Δ*V/*Δ*T*). By excluding the Seebeck voltage from the Pt lines, the Seebeck coefficient of the nanowire sample (*S*_nw_) was calculated as^[^ ^91^ ^]^ *S*_nw_ = *S*_measured_ + *S*_Pt_, where *S*_Pt_ is the Seebeck coefficient of Pt and was obtained based on the previously reported value.^[^ ^95^ ^]^ Electrical measurements were performed using a standard four‐point method. The electrical resistance was obtained by linear‐fitting the IV curve (Stanford Research, SR560; Keithley 6487).

### Magneto‐Transport Measurements

Magnetoresistance measurements were carried out in a partially home‐configured magneto‐transport measurement system that uses the Quantum Design Magnetic Property Measurement System (MPMS). Each device was secured onto the end of a long hollow rod and connected to a Linear Research 700 (LR‐700) AC Resistance Bridge via wires that were sent through the center of the rod. The resistance of the nanowire was then measured in a typical four‐terminal configuration with the magnetic field perpendicular to the nanowire axis. The temperature and magnetic field were set and read by the MPMS software while the values were simultaneously recorded in the same data file in which the resistance was recorded. The resistance values were averaged at each magnetic field point after the field was stable at the setpoint, which was read by the MPMS software.^[^ ^96^ ^]^

### Density Functional Theory Calculations

DFT calculations were performed with the projected‐augmented plane‐wave method,^[^ ^97^, ^98^ ^]^ as implemented in the Vienna ab initio Simulation Package (VASP).^[^ ^99^ ^]^ The exchange‐correlation of electrons was treated within the generalized gradient approximation in the form of Perdew–Berke–Ernzwehof (PBE).^[^ ^100^ ^]^ The convergence criterion for the forces acting on each atom was smaller than 0.001 eV Å^−1^ and the total energy was set to be 10^−6^ eV. A 12 × 12 × 8 Monkhorst–Pack k‐mesh was employed to sample the Brillouin zone. The energy cutoff for the plane‐wave‐basis expansion was set to 400 eV. The Hubbard *U* correction for V atoms (3.4 eV) was considered to describe strong electronic correlations.^[^ ^101^ ^]^ The SOC was included in the self‐consistent calculations because SOC effects were substantial for Ir.^[^ ^28^ ^]^ The Seebeck coefficients at different temperatures were calculated by solving the semiclassical Boltzmann transport equations within the constant relaxation time approximation (*τ* = 10^−14^ s) using the BoltzWann code^[^ ^102^ ^]^ implemented in WANNIER90 package.^[^ ^103^ ^]^ A dense 180 × 180 × 120 mesh was utilized to calculate the Seebeck coefficients. The phonon dispersion was calculated using the Phonopy code.^[^ ^104^ ^]^

## Conflict of Interest

The authors declare no conflict of interest.

## Supporting information

Supporting Information

## Data Availability

The data that support the findings of this study are available from the corresponding author upon reasonable request.
