# Supplementary material for: Enhanced Electron Correlation and Significantly Suppressed Thermal Conductivity in Dirac Nodal‐Line Metal Nanowires by Chemical Doping
Source: Adv Sci (Weinh). 2022 Nov 27;10(2):2204424. doi: 10.1002/advs.202204424 (PMC9839858; doi:10.1002/advs.202204424)
Supplement: Supplementary file 1 — Supporting Information [file ADVS-10-2204424-s001.pdf]

# Supporting Information

**Enhanced electron correlation and significantly suppressed thermal conductivity in Dirac nodal-line metal nanowires by chemical doping**

*Amanda L. Coughlin, Zhiliang Pan, Jeonghoon Hong, Tongxie Zhang, Xun Zhan, Wenqian Wu, Dongyue Xie, Tian Tong, Thomas Ruch, Jean J. Heremans, Jiming Bao, H. A. Fertig, Jian Wang, Jeongwoo Kim, Hanyu Zhu, Deyu Li,* and Shixiong Zhang**

## 1. Synthesizing V-doped $IrO_2$ ($Ir_{1-x}V_xO_2$) nanowires using a three-zone quartz tube furnace

$Ir_{1-x}V_xO_2$ nanowires were synthesized in a three-zone quartz tube furnace via a chemical vapor deposition (CVD) process [Figure S1]. Vanadium oxide ($VO_2$) powder (Aldrich, ≥99% trace metals basis) and iridium oxide ($IrO_2$) powder (Alfa Aesar, 99.99% metals basis) were placed in alumina boats in the center of zones 1 and 2, respectively, and silicon substrates were placed in the downstream zone 3. Prior to the growth, the Si substrates were scratched with a diamond tip pen to create a fresh and rough surface.

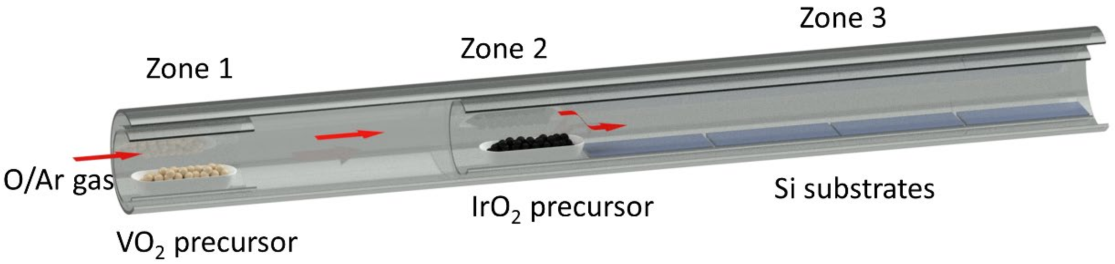


**Figure S1.** A schematic of the three-zone quartz tube furnace used to grow $Ir_{1-x}V_xO_2$ nanowires.

## 2. Density contrast of nanowires varies with the depth of the scratches and the soaking time

As shown in Figure S2, the contrast in the density of nanowires in the scratched versus unscratched regions becomes less dramatic with an increase in growth duration. A longer growth time may lead to more nucleation occurring and thus more nanowires are grown on the flat regions of the substrate. Additionally, the depth of the scratches has an impact on the density of nanowires grown on the substrates. In the order of the deepest to shallowest scratches: Figure S2(b) > (a) > (c), where there is a clear decrease in density of nanowires in the scratches as the depth of the scratches decreases.

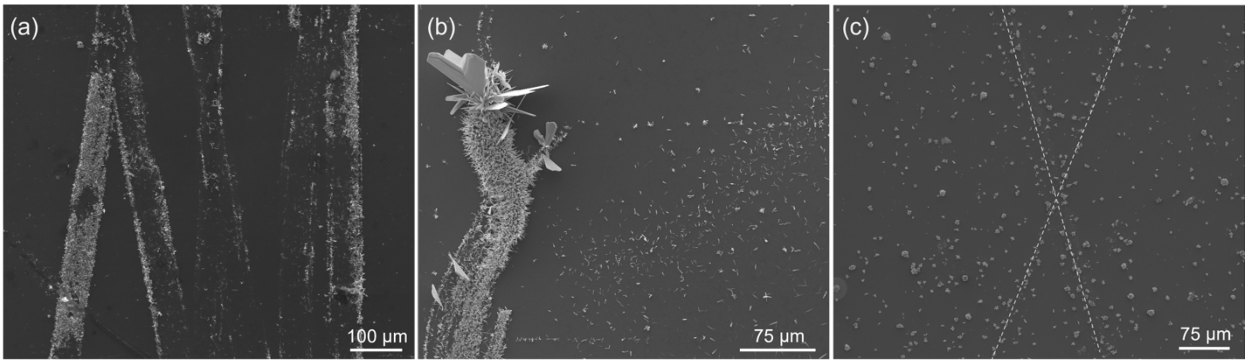


**Figure S2.** SEM images showing a higher density of nanowires growing out of the scratched regions of the substrates in which either a (a) 30 min, (b) 120 min, and (c) 300 min soak time was used during the growths, respectively. We note that the scratches in the 120 min growth were the deepest between the three growths. The dashed X in (c) is used as a guide to the eye to indicate the shallow scratches on the substrate.

### 3. Tapered morphology of nanowires outside of the scratches

On the unscratched regions of the growth substrates, the diffused atoms wet on the flat substrate and are more inclined to grow along the substrate and are typically partially embedded in the substrate, leading to a more tapered-looking morphology, as shown in Figure S3.

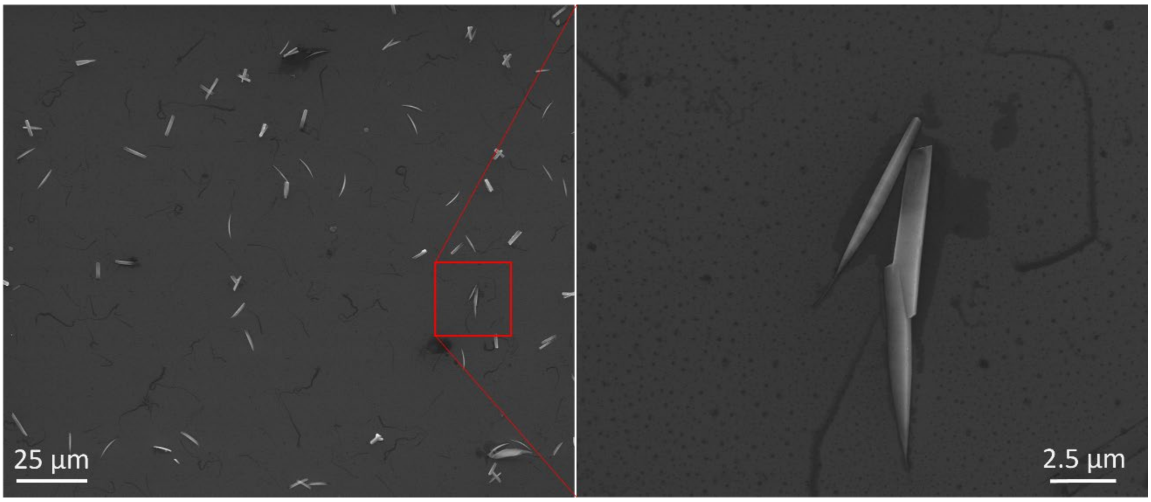


**Figure S3.** SEM images of nanowires grown on a flat, unscratched region of a growth substrate with a tapered-looking morphology.

## 4. TEM-XEDS measurements of oxygen with a single detector versus dual detectors

The TEM-XEDS oxygen mapping of a doped $IrO_2$ nanowire measured with a single detector appears asymmetric across its diameter [Figure S4], however, this effect is due to the absorption effect when only one detector is positioned asymmetrically from the nanowire (*e.g.,* to the left or right of the nanowire). This result can be corrected with a measurement using a dual-detector system, as shown in Figure S5, where the oxygen mapping is shown to be symmetric.

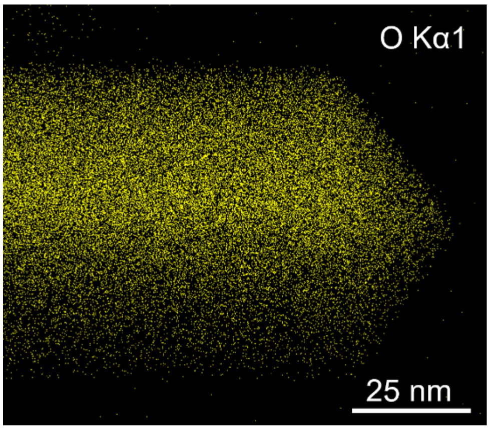


**Figure S4.** The asymmetric TEM-XEDS mapping of O measured with a single detector.

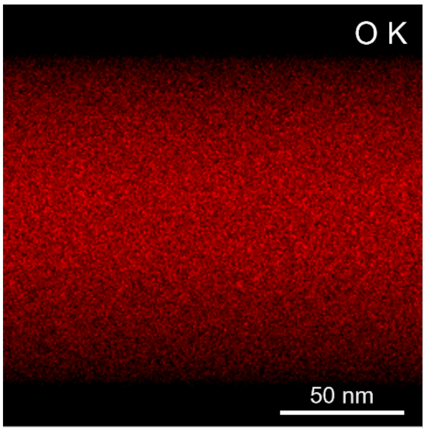


**Figure S5.** The symmetric TEM-XEDS of the O concentration in an $IrO_2$ nanowire measured with a dual-detector.

## 5. Measuring the V concentration of nanowires in Raman measurements via SEM-XEDS

The V concentration of the nanowires in the Raman measurements ($x = \frac{V}{V+Ir}$) were measured through SEM-XEDS. As shown in Figure S6, the nanowire has ~16% V, where we estimate the error to be within a few percent for this semi-quantitative tool.

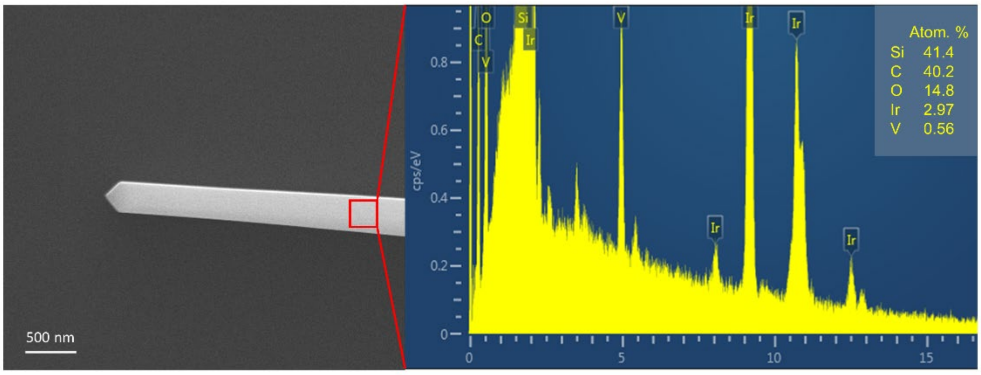


**Figure S6.** A SEM image and XEDS spectrum of a representative nanowire.

## 6. Raman measurements and peak fittings on nanowires of various V concentrations

A laser power of 50 µW was used for all Raman measurements and all of the nanowires measured were of similar diameters (~320 nm). The Raman peaks for each spectrum were fitted [Figure S7] using IGOR Pro 6.37's built in multi-peak fitting package which provides quantities for the amplitude, area intensity, FWHM, etc. and their respective errors.

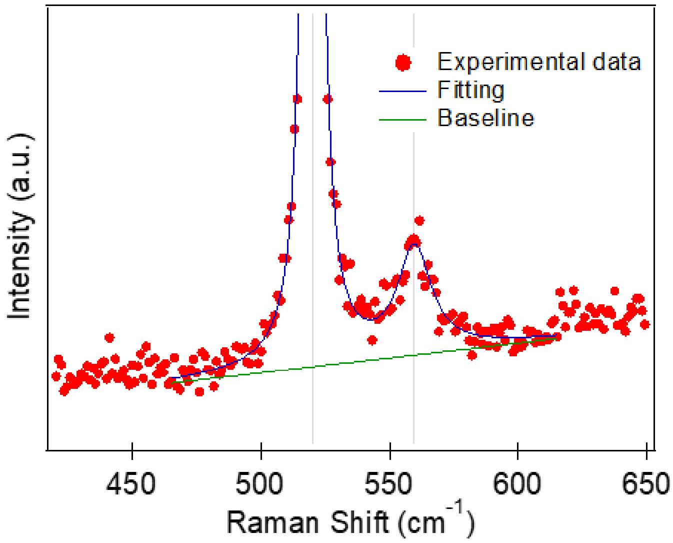


**Figure S7.** A fitting of the 520 cm$^{-1}$ (Si substrate) and ~560 cm$^{-1}$ peaks in the spectra of a representative $Ir_{1-x}V_xO_2$ nanowire in the range between ~465 and 615 cm$^{-1}$.

## 7. Measuring the V concentration of nanowires via TEM-XEDS

After the transport measurements, we transferred the same nanowires to a TEM grid and performed TEM-XEDS on three different regions of each nanowire (near each of the ends and a region in the middle) and averaged them to determine their respective V concentrations ($x$), as shown in Figure S8. The average $x$ values for the nanowires of various diameters are shown in Table S1.

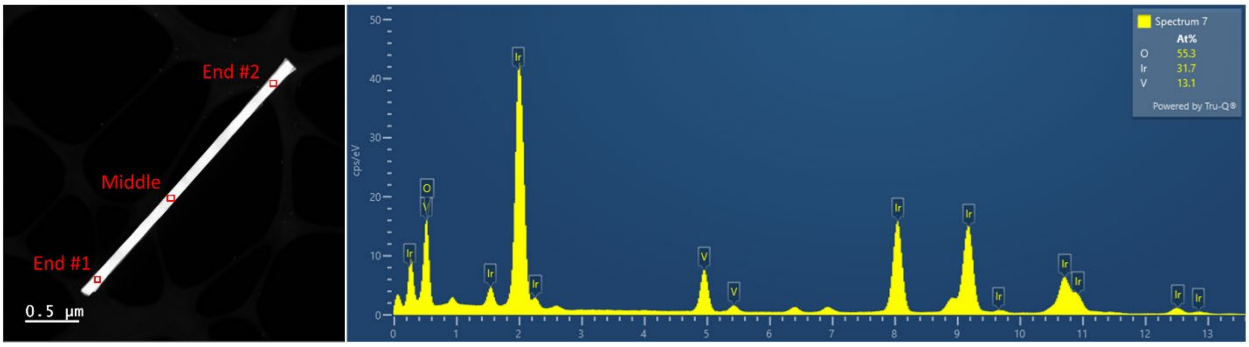


**Figure S8.** A TEM image of a representative $Ir_{1-x}V_xO_2$ nanowire indicating the three regions where TEM-XEDS spectra were measured and the respective XEDS spectra from the middle region.

| **Diameter (nm)** | 87 | 108 | 139 | 143 | 149 |
|---|---|---|---|---|---|
| **Average *x*** | 0.29 | 0.28 | 0.30 | 0.29 | 0.29 |

**Table S1.** The diameter and respective averaged $x$ values determined from TEM-XEDS.

## 8. The electronic density of states of $IrO_2$ and $V_{0.25}Ir_{0.75}O_2$

The electronic density of states for $IrO_2$ and $Ir_{0.75}V_{0.25}O_2$ were calculated using density functional theory, as shown in the top and bottom panels of Figure S9, respectively.

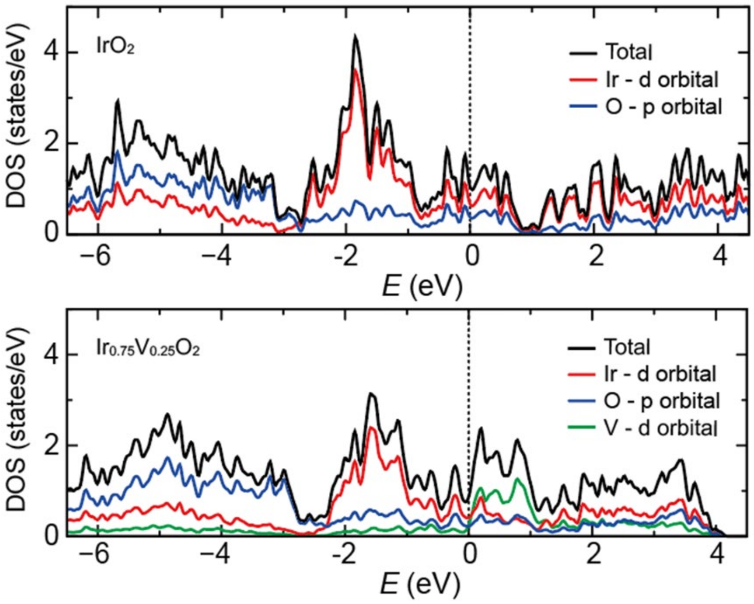


**Figure S9.** DFT-calculated density of states in $IrO_2$ (top panel) and $V_{0.25}Ir_{0.75}O_2$ (bottom panel).

## 9. The Analysis of electrical transport data using thermal activation model

The expression for electrical resistivity $\rho$ resulting from activation across a bandgap, such as in semiconductors, is written as: $\rho = e^{\frac{E_g}{2k_BT}}$, where $E_g$ is the bandgap and $k_B$ is the Boltzmann constant. If it is thermal activation which leads to the non-metallic behavior, $\ln\rho$ would be proportional to $T^{-1}$, which is not the case for our samples [Figure S10].

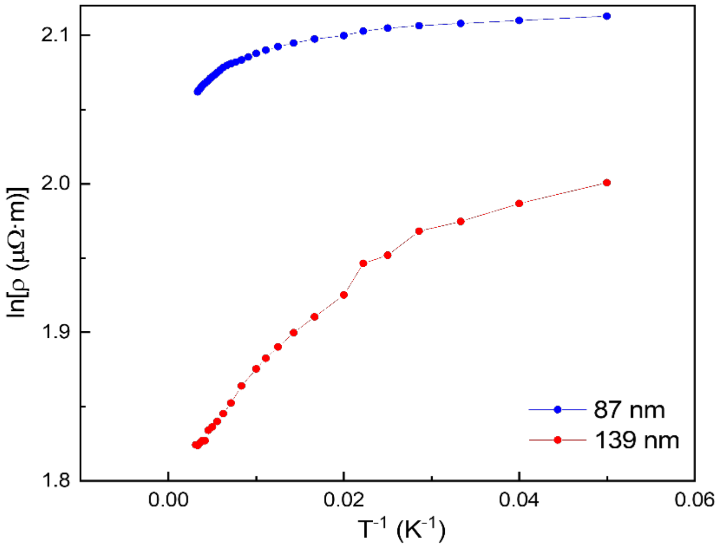


**Figure S10.** Plot of $\ln\rho$ vs. $T^{-1}$, the nonlinear behavior rules out thermal activation

## 10. The electronic band structures of $IrO_2$ and $Ir_{0.75}V_{0.25}O_2$

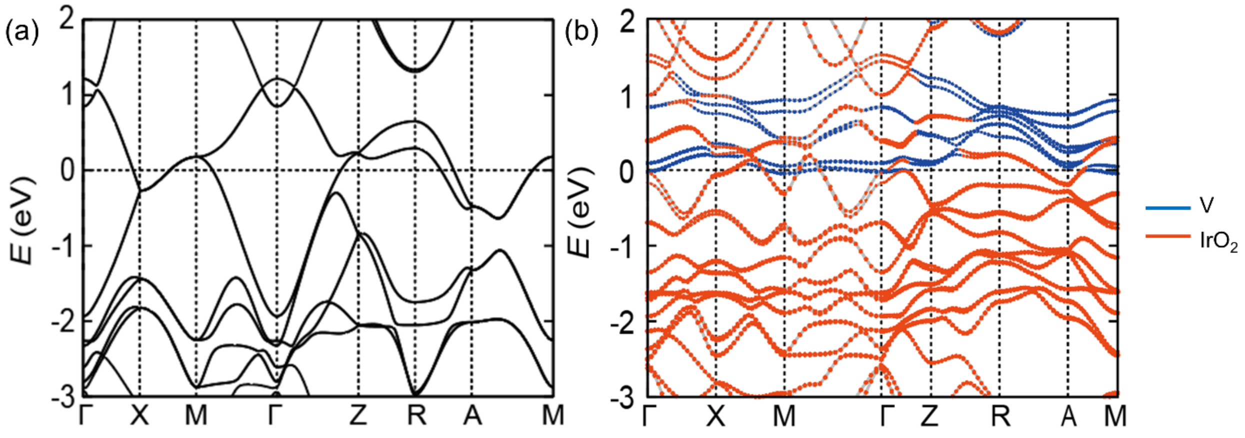


**Figure S11.** The electronic band structures of (a) undoped $IrO_2$ and (b) $Ir_{0.75}V_{0.25}O_2$.

**11. Analysis of electrical transport data using Anderson localization**

The behavior of resistivity caused by Anderson (strong) localization is described by the variable range hopping (VRH) model: $\rho = \rho_0 \cdot \exp\left(\frac{T_0}{T}\right)^{1/(v+1)}$, where $v$ is the dimensionality. By fitting the data in the low temperature regime, we can find $T_0$ and the localization length $\xi$ can be computed using $T_0 = \frac{16}{k_B \cdot N(E_F) \cdot \xi^3}$,[1] where the density of states at the Fermi energy $N(E_F)$ can be determined from Figure S9. Figure S12 shows the fitting of the 87 nm and 139 nm nanowires when $v = 3$. The fitting parameters and determined localization lengths for all dimensionalities are displayed in Table S2. The 139 nm nanowire is expected to have the smallest localization length since it has the largest change of resistivity, however, its localization length is still much larger than the size of unit cell. As for the other nanowires, $\xi$ are found to be hundreds of times the size of the unit cell. Therefore, it is unlikely that Anderson localization plays an important role in the behavior of resistivity.

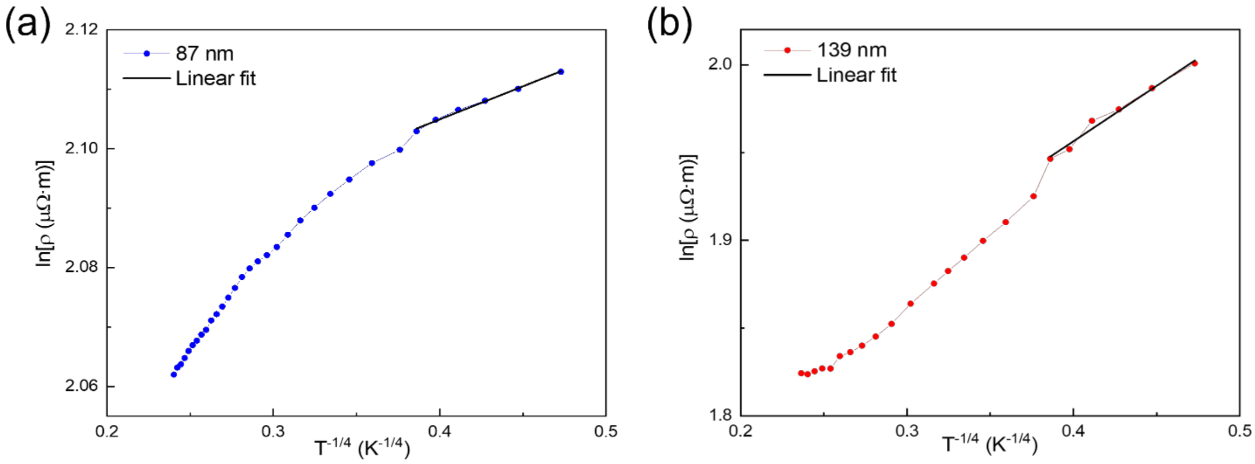


**Figure S12.** Plot of ln$\rho$ vs. $T^{-1/4}$. 3D VRH model is applied in the range of 20-45 K, and $T_0$ is determined by the slope.

(a)

| 87 nm | $v = 1$ | $v = 2$ | $v = 3$ |
| --- | --- | --- | --- |
| slope | 0.129 | 0.110 | 0.111 |
| $T_0(K)$ | 0.017 | 0.0013 | 0.00015 |
| $\xi$ (Å) | 737 | 1737 | 3569 |
| # unit cell = $\frac{\xi}{a}$ | 164 | 386 | 793 |

(b)

| 139 nm | $v = 1$ | $v = 2$ | $v = 3$ |
| --- | --- | --- | --- |
| slope | 0.733 | 0.627 | 0.631 |
| $T_0(K)$ | 0.537 | 0.246 | 0.159 |
| $\xi$ (Å) | 233 | 303 | 350 |
| # unit cell = $\frac{\xi}{a}$ | 52 | 67 | 78 |

**Table S2.** The fitting parameters and localization lengths for the (a) 87 nm and (b) 139 nm nanowires.

**12. Magnetoresistance (MR)**

Magnetoresistance data were taken at temperatures between 20-200 K and 5-15 K while sweeping the field from -6.5 T to 6.5 T and from -1 T to 1 T, respectively, as shown in Figure S13. The MRs observed at all temperatures just fluctuate around 0 with an order of 0.1%, showing no specific pattern. Although the MR data do not show any localization or anti-localization mechanism, the absence of any obvious magnetic field effect is expected of the orbital 2-channel Kondo effect.[2]

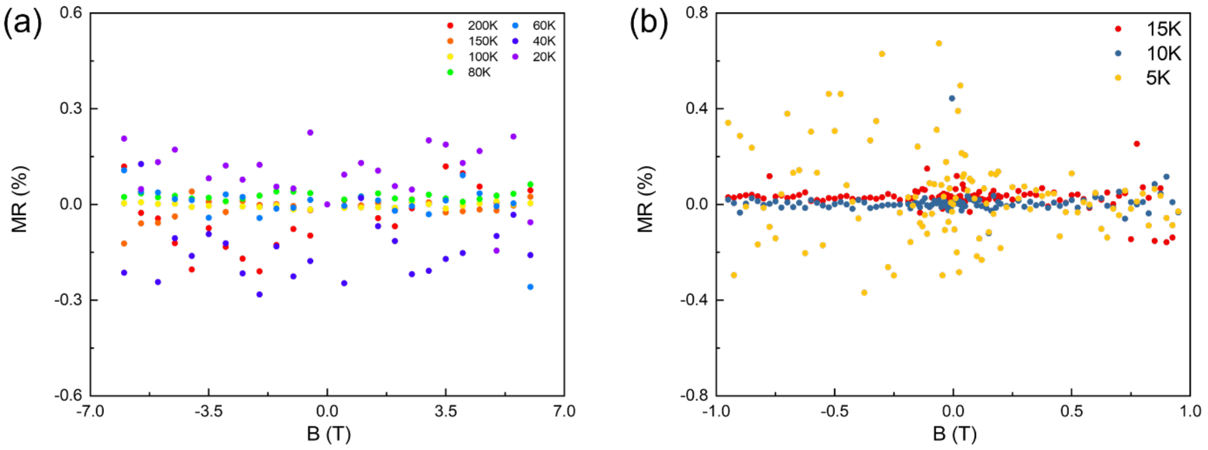


**Figure S13.** Magnetoresistance data taken at different temperatures (a) between 20-200 K and (b) between 5-15 K.

**13. Analysis of electrical transport data using the direct electron-electron interaction effect (EEI)**

The direct EEI effect is a candidate to explain the nonmetallic behavior of $IrO_2$ nanowires. For an isotropic 3D system, the direct EEI effect is described by:[2-6]

$$\frac{\Delta\rho(T)}{\rho(T_0)} = -\frac{0.915e^2}{4\pi^2\hbar}\left(\frac{4}{3}-\frac{3}{2}\tilde{F}\right)\rho(T_0)\sqrt{\frac{k_B}{\hbar D}}\left(\sqrt{T}-\sqrt{T_0}\right) \qquad (1)$$

Where $\Delta\rho(T) = \rho(T) - \rho(T_0)$, $T_0$ is the reference temperature, and $\tilde{F}$ is the screening factor. $D$ is the diffusion constant and can be computed using $D = \frac{1}{\rho e^2 N(E_F)}$, where $\rho$ is the resistivity. To explore the existence of the direct EEI effect, we analyze the 139 nm nanowire which has the largest change in resistivity. Choosing $T = 20\ K$ and $T_0 = 30\ K$ from the experimental data, we calculate $\frac{\Delta\rho(T)}{\rho(T_0)} = 2.65\%$, while the Eq. (1) predicts the largest possible increase is 0.35% with $\tilde{F} = 0$. Similarly, we perform the analysis on the 108 nm and 87 nm nanowires (the data on the 149 nm and 143 nm nanowires are encumbered by noise), and find the increases are 0.24% and 0.49% respectively, while the corresponding predicted values are 0.36% and 0.43%. While the data on the 139 nm nanowire shows some deviations from the predicted value, the 108 nm and 87 nm nanowires agree with the prediction, both showing increases less than the possible largest value. Hence the direct EEI effect cannot readily be excluded as a viable explanation.

**14. Estimation of mean free path and electron-phonon scattering rate**

To estimate the mean free path, we use the equation $l = \frac{3}{v_F \rho e^2 N(E_F)}$, where $v_F$ is the Fermi velocity, $\rho$ is the resistivity and $N(E_F)$ is the density of states at Fermi energy. The $v_F$ estimated from the DFT calculated band structure is approximately $10^5$ m/s. Together with the $N(E_F) \sim 1.7 \times 10^{47}\ m^{-3} J^{-1}$ and $\rho(20K) \sim 7 \times 10^{-6}\ \Omega m$ extracted from the DFT calculation and experimental data, respectively, we have estimated the mean free path to be $l(20\ K) \sim 1$ nm, which is of the same order, but smaller, than the $l(10\ K) = 2.5 - 3.7$ nm of $IrO_2$ calculated in the literature due to the doping enhanced scattering and higher temperature.[2]

Electron-phonon (e-ph) scattering usually reveals itself by showing a $\rho \propto T^5$ behavior at low temperatures. However, this temperature dependence is not observed in our system because of the weaker contribution of electron-phonon scattering in comparison to the other mechanisms discussed in our paper. The absence of this dependence makes it difficult to extract information about e-ph scattering directly from the electrical transport data. On the other hand, since we observed a clear size effect in lattice thermal conductivity, a rough estimation is that the e-ph scattering rate is comparable to or weaker than the boundary scattering. Our DFT calculations of phonon dispersion suggests that the acoustic phonon group velocity is on the order of kilometers per second, and the nanowires are around 100 nm wide. We then roughly estimate the rate of phonon-boundary scattering rate to be $\tau_{ph-boundary}^{-1} \approx \frac{v_{group}}{D_{wire}} \sim 10^{10}\ \mathrm{s}^{-1}$ and the electron-phonon scattering rate to be $\tau_{e-ph}^{-1} \leq 10^{10}\ \mathrm{s}^{-1}$. Combined with the Fermi velocity, we estimate that the characteristic length scale of e-ph scattering is on the order of a micron, which is much larger than the electron mean free path, indicating the contribution of e-ph scattering to the charge transport is indeed negligible in our system.

**15. Analysis of electrical transport data using weak localization (WL)**

Weak localization is another mechanism which can lead to a mild increase of resistivity with decreasing temperature [Figure 3(c)]. WL is due to coherent backscattering on time-reversed paths and is sensitive to the quantum phase coherence length which itself lengthens with decreasing temperature.[3, 4, 6-15] As a result, localization increases as temperature decreases. A dominant mechanism affecting the quantum coherence length is Nyquist dephasing, where an electron experiences the fluctuating electromagnetic environment due to all other electrons, leading to quasi-elastic EEI effects (involving small energy transfer).[3, 4, 6-8, 10-15] In a wire, due to Nyquist dephasing we expect a $T^{-p}$ ($1/3 < p < 1/2$) correction to the resistivity (increasing with decreasing temperature), with $T^{-1/3}$ in strictly 1D[7, 8, 10, 13, 15] and $T^{-1/2}$ in strictly 2D.[3, 7, 12, 14, 15] To test the WL model, we fit the resistivity data of the 87 nm nanowire using $\rho = A + BT^{-p}$ where $p$ is positive. From Figure S14, $p$ is found to be 0.02, which indicates WL is unlikely to be a dominant effect on the transport in this sample.

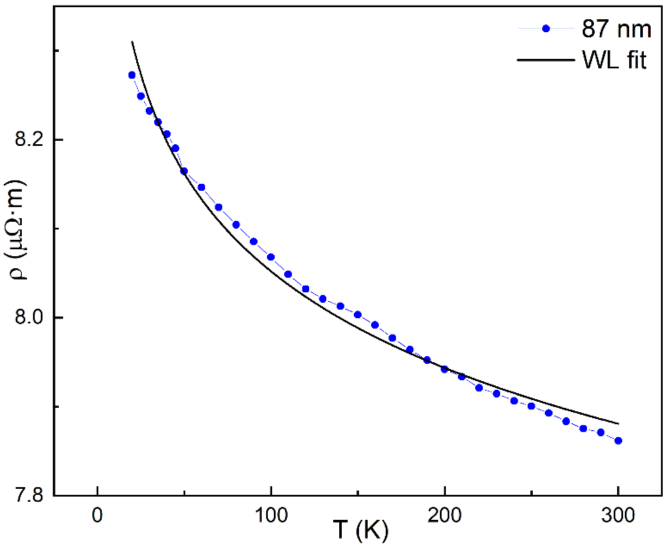


**Figure S14.** Using the WL model to fit the resistivity data of the 87 nm wire.

**16. Contact thermal resistance after wetting and EBID treatment is negligible**

The effect of the contact thermal resistance was estimated by measuring the same nanowire with different contact treatments. As shown in Figure S15, the measured thermal conductance increases after the wetting treatment and the first round of EBID but remains essentially the same after a second round of EBID. As such, the contact thermal resistance after wetting and EBID treatment can be regarded as negligible.

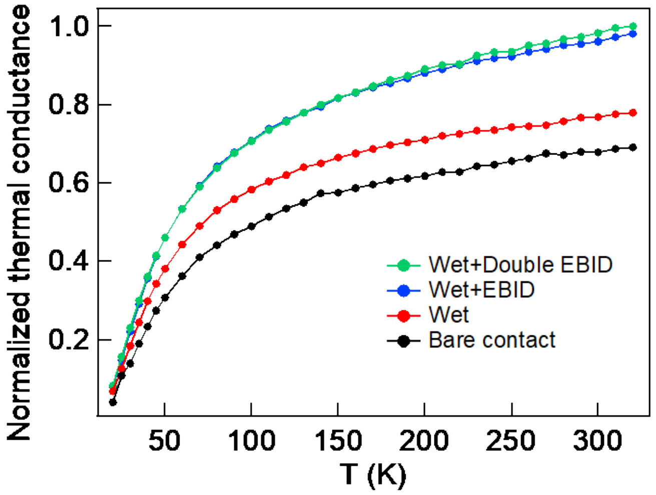


**Figure S15.** Normalized thermal conductance of the same wire with different contact treatments: wetting + double EBID; wetting + EBID; wetting; bare contact.

**17. Diameter-dependent thermal conductivity in the $Ir_{1-x}V_xO_2$ nanowires**

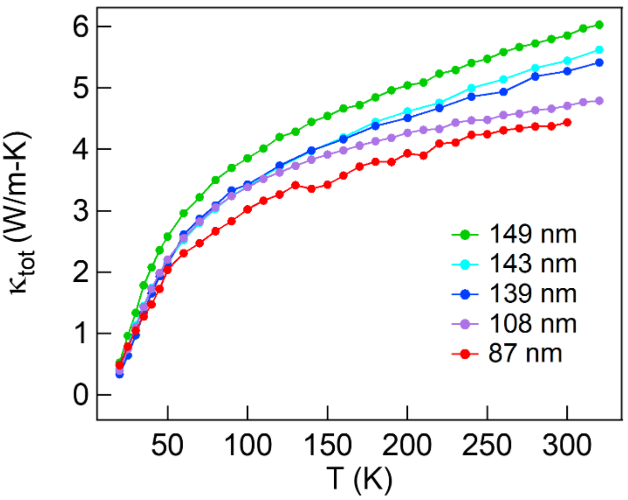


**Figure S16.** The total thermal conductivity of $Ir_{1-x}V_xO_2$ nanowires of different sizes.

**References**


[1] Mott, N. F.; Davis, E. A., *Electronic Processes in Non-Crystalline Materials*, Oxford university press, **2012**.
[2] Yeh, S. S.; Su, T. K.; Lien, A. S.; Zamani, F.; Kroha, J.; Liao, C. C.; Kirchner, S.; Lin, J. J., *Nat. Commun.* **2020**, *11*, 1.
[3] Akkermans, E.; Montambaux, G., *Mesoscopic Physics of Electrons and Photons*, **2007**.
[4] Lee, P. A.; Ramakrishnan, T., *Rev. Mod. Phys.* **1985**, *57*, 287.
[5] Altshuler, B.; Khmel'Nitzkii, D.; Larkin, A.; Lee, P., *Phys. Rev. B.* **1980**, *22*, 5142.
[6] Barone, C.; Rotzinger, H.; Mauro, C.; Dorer, D.; Münzberg, J.; Ustinov, A.; Pagano, S., *Sci. Rep.* **2018**, *8*, 1.
[7] Xie, Y.; Heremans, J., *Phys. Rev. B.* **2018**, *98*, 035429.
[8] Kallaher, R.; Heremans, J. J.; Goel, N.; Chung, S.; Santos, M., *Phys. Rev. B.* **2010**, *81*, 035335.
[9] Kallaher, R.; Heremans, J. J.; Van Roy, W.; Borghs, G., *Phys. Rev. B.* **2013**, *88*, 205407.
[10] Rudolph, M.; Heremans, J. J., *Phys. Rev. B.* **2011**, *83*, 205410.
[11] Lin, J.-J.; Bird, J., *J. Phys.: Condens. Matter.* **2002**, *14*, R501.
[12] Treiber, M.; Texier, C.; Yevtushenko, O. M.; von Delft, J.; Lerner, I. V., *Phys. Rev. B.* **2011**, *84*, 054204.
[13] Natelson, D.; Willett, R.; West, K.; Pfeiffer, L., *Phys. Rev. Lett.* **2001**, *86*, 1821.
[14] Choi, K.; Tsui, D.; Alavi, K., *Phys. Rev. B.* **1987**, *36*, 7751.
[15] Altshuler, B. L.; Aronov, A.; Khmelnitsky, D., *Journal of Physics C: Solid State Physics.* **1982**, *15*, 7367.
